# *Lactiplantibacillus plantarum* MG4296 and *Lacticaseibacillus paracasei* MG5012 Ameliorates Insulin Resistance in Palmitic Acid-Induced HepG2 Cells and High Fat Diet-Induced Mice

**DOI:** 10.3390/microorganisms9061139

**Published:** 2021-05-25

**Authors:** Gayeong Won, Soo-Im Choi, Chang-Ho Kang, Gun-Hee Kim

**Affiliations:** 1 Department of Health Functional New Materials, Duksung Women’s University, Seoul 01369, Korea; gywon04@gmail.com; 2 Mediogen, Co., Ltd., Seoul 04146, Korea; changho-kang@naver.com; 3 Department of Food and Nutrition, Duksung Women’s University, Seoul 01369, Korea

**Keywords:** *Lactiplantibacillus plantarum* MG4296, *Lacticaseibacillus paracasei* MG5012, insulin resistance, diabetes, PI3K/Akt

## Abstract

The purpose of this study was to evaluate the capacity of *Lactiplantibacillus plantarum* MG4296 (MG4296) and *Lacticaseibacillus paracasei* MG5012 (MG5012) on insulin resistance (IR) and diabetes-related metabolic changes in palmitic acid (PA)-induced HepG2 cells and high-fat diet-induced mice. In vitro, cell-free extracts of MG4296 and MG5012 alleviated IR by increasing glucose uptake and glycogen content in PA-induced insulin-resistant HepG2 cells. In vivo, MG4296 and MG5012 supplementation markedly decreased body weight and glucose tolerance. Administration of both strains also improved serum glucose, glycated hemoglobin, insulin, triglyceride, LDL/HDL ratio, and homeostatic model assessment of IR (HOMA-IR). Histopathological analysis of liver tissue demonstrated a significant reduction in lipid accumulation and glycogen content. Moreover, MG4296 and MG5012 treatment enhanced phosphoinositide-3 kinase (PI3K)/protein kinase B (Akt) expression in the liver. Overall, MG4296 and MG5012 could prevent HFD-induced glucose tolerance and hyperglycemia by improving IR. Therefore, *L. plantarum* MG4296 and *L. paracasei* MG5012 could be useful as new probiotics candidates to improve T2DM.

## 1. Introduction

Diabetes mellitus (DM), a representative metabolic disease, is recognized as a major risk factor in the deterioration of human health worldwide, along with obesity [1]. DM is characterized by symptoms of hyperglycemia. More than 90% of DM patients are classified as type 2 DM (T2DM) [2]. T2DM is caused by a complex etiology characterized by resistance to insulin action, inadequate insulin secretion response, excessive glucose production in the liver, insulin resistance (IR), and decreased glucose processing capacity of muscle and fat cells [3]. In T2DM, IR can be defined as a metabolic state in which insulin action (insulin sensitivity) is relatively lower given the normal state of physiological insulin concentrations [4,5]. IR occurs in target organs, such as the liver, muscle, and adipose tissues (AT) [6]. IR causes compensatory hyperinsulinemia with metabolic abnormalities, such as glucose hypersensitivity, hyperlipidemia, hepatic steatosis, and nonalcoholic fatty liver disease (NAFLD) [7]. The phosphoinositide-3 kinase (PI3K)/protein kinase B (Akt) pathway plays an important role in regulating IR and glucose metabolism [8]. Tyrosine phosphorylation of the insulin receptor substrate (IRS) in response to insulin stimulation activates PI3K [9]. Activation of PI3K catalyzes the phosphorylation of Akt to activate downstream proteins, thereby increasing glycogen synthesis [10].

Gut microbial communities are known to significantly impact the host’s physiology and pathology and play an important role in the digestion and extraction of nutrients, modification of the host immune response, prevention of infection, and metabolism of drugs [11]. It is known that intestinal microflora is affected by various environmental factors, including food and probiotics consumption [12]. The Food and Agriculture Organization defines probiotics as living microbial strains that provide health benefits to the host when administered in the proper amount [13]. Probiotics were found to be effective against irritable bowel syndrome, inflammatory bowel disease, diarrhea, atopic dermatitis, and metabolic diseases [14]. Numerous studies have demonstrated that probiotics are associated with regulating glycemic metabolism, but the potential mechanism has not been fully elucidated. The proposed antidiabetic mechanisms of probiotics are an improvement in carbohydrate metabolism by increased secretion of glucagon-like peptide-1 in intestinal endocrine L cells [15], an increase in insulin sensitivity by lipid profile improvement [16], limiting of carbohydrate hydrolysis and absorption by α-glucosidase inhibition [17], antioxidative activity [18], anti-inflammatory activity [19], and changes in diabetes-related genes [20]. Recently, various studies have demonstrated that changes in gut microbiota are associated with IR and the development of T2DM [21,22,23]. In particular, short-chain fatty acids (SCFAs), such as acetate, propionate, and butyrate, produced during the decomposition of carbohydrates in the gut microbiota, act as an energy source for intestinal epithelial cells, strengthen the immune system, reduce inflammation, and regulate metabolism [24].

Our previous study identified and selected two potential probiotic strains with hypoglycemic and antioxidative effects in vitro. Therefore, this study aimed to investigate the antidiabetic effect of *Lactiplantibacillus plantarum* MG4296 and *Lacticaseibacillus paracasei* MG5012 and demonstrate the underlying molecular mechanisms associated with glucose metabolism in HepG2 cells in vitro and in a high-fat diet and sugar water-induced T2DM mouse model.

## 2. Materials and Methods

### 2.1. Materials

Lactobacilli de Man, Rogosa, and Sharpe (MRS) agar and broth used for the cultivation and analysis of lactic acid bacteria (LAB) were purchased from Difco (Detroit, MI, USA). Caco-2 and HepG2 cells were obtained from the Korean Cell Line Bank (Seoul, Korea). Minimum essential medium (MEM), Dulbecco’s modified Eagle’ s medium (DMEM), fetal bovine serum (FBS), penicillin–streptomycin (P/S), and phosphate-buffered saline (PBS) were purchased from Gibco (Gaithersburg, MD, USA). Antibodies against p-IRS-1 (Ser 307), IRS-1, p-PI3K (Tyr 485), PI3K, p-Akt (Ser 473), Akt, and GAPDH were purchased from Cell Signaling Technology (Danvers, MA, USA). Bovine serum albumin (BSA), dimethyl sulfoxide (DMSO), radioimmunoprecipitation (RIPA) buffer, 2-Deoxy-2-[(7-nitro-2,1,3-benzoxadiazol-4-yl) amino]-D-glucose (2-NBDG), and all other reagents used in the experiments were purchased from Sigma-Aldrich (St. Louis, MO, USA).

### 2.2. Sample Preparation

*Lactiplantibacillus plantarum* MG4296 (MG4296) and *Lacticaseibacillus paracasei* MG5012 (MG5012) were supplied by MEDIOGEN Co., Ltd. (Jecheon, Korea). Each strain was activated by culturing in MRS medium at 37 °C for 18 h.

The cells were collected by centrifugation (15,000× *g*, 15 min, 4 °C) and washed three times with PBS to remove the remaining broth to prepare for the in vitro study. The cells were resuspended in PBS and adjusted to 10^9^ CFU/mL. The bacterial cells were placed in an ice bath and then ultrasonicated for 30 min. The pulverized cells were filtered through a 0.2 μm syringe filter to prepare cell-free extracts (CFEs). Samples were stored at −70 °C until use.

For the in vivo study, freshly harvested bacterial pellets were mixed with the cryoprotectant mixture (equal amounts of sodium alginate and pumpkin powder) in a 1:2.5 (*w*/*w*) ratio [25]. The bacterial cells were dispersed in a stainless still container and freeze-dried (Heto Dry winner, Allerod, Denmark). Primary drying was performed to a shelf temperature of −40 °C for 1 h. Secondary drying was performed stepwise up to 20 °C for a total of 24 h. The powdered cells were harvested, collected in polythene bags, wrapped in aluminum foil, and stored at 4 °C until further use.

### 2.3. Cell Viability in HepG2 Cells

HepG2 cell viability was measured using a thiazolyl blue tetrazolium bromide (MTT) assay. HepG2 cells were cultured at 2 × 10^4^ cells/well in a 96-well plate and treated with different concentrations of CFEs of MG4296 or MG5012 (10^7^–10^9^ CFU/mL) for 24 h. Subsequently, the MTT solution (5 mg/mL) was added to each well and incubated at 37 °C for 4 h. After removing this medium, 100 μL of DMSO was added to dissolve the formazan crystals. After shaking, absorbance was measured at 540 nm using a microplate reader (Spectra Max 190, Molecular Devices, San Jose, CA, USA).

### 2.4. Cell Culture and Treatment

A palmitic acid (PA)-induced insulin-resistant HepG2 cell model was estimated as described by Zhang et al. [26]. Briefly, HepG2 cells were cultured in DMEM supplemented with 10% FBS (*v*/*v*) and 1% P/S (*v*/*v*) at 37 °C in a 5% CO_2_ atmosphere. The PA solution was prepared in 100 mM sodium hydroxide at 70 °C and then diluted in 10% (*w*/*v*) BSA solution. After reaching 80–90% confluence, the cells were treated with PA (0.25 mM) in the absence or presence of CFEs for 24 h to induce an in vitro IR model.

### 2.5. Glucose Uptake

Glucose uptake in PA-induced HepG2 cells was estimated using 2-NBDG according to Yan et al. [27]. HepG2 cells were cultured at 2 × 10^5^ cells/well in 24-well plates and treated with PA in the presence of CFEs for 24 h. The cells were then stored in glucose-free DMEM for 4 h and then stimulated with 0.5 mM insulin for 10 min. After incubation at 37 °C with 0.05 μM 2-NBDG in DMEM without glucose for 30 min, the cells were washed twice with cold PBS to terminate the reaction. Fluorescence was detected at an excitation wavelength of 485 nm and an emission wavelength of 535 nm.

### 2.6. Glycogen Contents

HepG2 cells were cultured at 5 × 10^5^ cells/well in a 6-well plate and grown to 80–90% confluence. CFEs were then added to the PA-induced IR HepG2 cells, and the cells were incubated for 24 h and then homogenized in 100 μL of water on ice. The homogenates were boiled for 5 min and centrifuged at 13,000× *g* for 5 min. The glycogen content in the homogenates was measured using a quantification kit (Sigma-Aldrich, St. Louis, MO, USA).

### 2.7. Animals and Experimental Design

C57BL/6 mice (six-week-old male, 18–20 g) were obtained from Orient-Bio Inc. (Seongnam, Korea). Mice were housed in an air-conditioned room at 23 ± 1.0 °C, with a relative humidity of 45 ± 5% under a 12-h light/dark cycle. The mice were provided with standard laboratory chow and water ad libitum. All procedures were handled according to the Guide of the Care and Use of Laboratory Animals (Institute of Laboratory Animal Resources, Commission on Life Sciences, National Research Council). The experimental protocols were approved by the Institutional Animal Care and Use Committee of Duksung Women’s University (2019-003-010), and every effort was made to minimize suffering. For the induction of T2DM, the mice were randomly divided into the following four groups (*n* = 6/group): (1) normal diet (ND), (2) high-fat diet (HFD), (3) HFD + MG4296, and (4) HFD + MG5012. Dosages of these strains were established at 1 × 10^9^ CFU/mouse based on in vitro studies. To induce significant obesity and IR in the mice, all HFD groups were fed a diet containing 60% fat, 20% protein, and 20% carbohydrates, and additionally, the mice were provided drinking water containing 42 g/L sugar (55% fructose/45% sucrose) [28]. The ND group was supplied a diet containing 14% fat, 21% protein, and 64% carbohydrates. The strain powder was dissolved in sterile PBS and orally administered to mice once daily for 12 weeks with an HFD supply.

### 2.8. Body Weight, Food Intake, Weight of Liver, and Adipose Tissues (AT)

Body weight and food intake were recorded once a week for 12 weeks. Mice were fed at the same time each day, and intake was calculated from the amount of food remaining. After the experiment and fasting for 12 h, mice in all groups were anesthetized by CO_2_ inhalation and euthanized by cardiac puncture.

The liver and AT (epididymal, mesenteric, perirenal, retroperitoneal, and subcutaneous) were dissected, rinsed with PBS buffer, weighed, and visually inspected. Extracted tissues were frozen in liquid nitrogen and stored at −70 °C until further analysis.

### 2.9. Oral Glucose Tolerance Test

During the last week of the experimental period, the mice were subjected to an oral glucose tolerance test (OGTT). Briefly, after fasting for 12 h, glucose (2 g/kg body weight) was orally administered to the mice. Thereafter, the blood glucose level was measured at 0, 15, 30, 60, 90, and 120 min by tail vein puncture using a glucose meter (Accu-Chek Performa, Roche, Mannheim, Germany). The area under the curve (AUC) of blood glucose levels was calculated to evaluate glucose tolerance.

### 2.10. Insulin Concentration and HOMA-IR

The serum insulin concentration was enzymatically quantified using a commercial ELISA kit (Invitrogen, Carlsbad, CA, USA). Homeostasis model for the IR index (HOMA-IR) was calculated using the following formula: HOMA-IR = [fasting glucose (mg/dL) × fasting insulin (μIU/mL)]/405

### 2.11. Serum Biochemical Analysis

Blood samples were collected into EDTA-coated tubes and placed at room temperature for at least 30 min. The serum was obtained by centrifuging at 3000 rpm for 20 min at 4 °C. The serum levels of aspartate aminotransferase (AST), alanine aminotransferase (ALT), total cholesterol (TC), triglyceride (TG), high-density lipoprotein cholesterol (HDL), and low-density lipoprotein cholesterol (LDL) were measured using a chemistry analyzer (Beckman Coulter, Brea, CA, USA). The glycated hemoglobin (HbA1c) concentration was enzymatically quantified using a commercial ELISA kit (Bio-Vision, Milpitas, CA, USA).

### 2.12. Histopathological Examination

The liver tissue was fixed in 10% formalin for at least 24 h, embedded in paraffin, sectioned at 4 μm, and stained with hematoxylin and eosin (H&E). Slides were subjected to microscopic examination. Hepatic steatosis was evaluated numerically according to semi-quantitative pathological criteria [29]. Histopathological scores for hepatic steatosis were quantified and expressed simply by the summation of individual 4-grades (score 0–3) as follows: 0 (<5%), 1 (5–33%), 2 (33–66%), and 3 (>66%). A board-certified toxicologic pathologist blindly performed all histological evaluation procedures.

### 2.13. Glycogen and TG Contents in Liver Tissue

To measure the glycogen content, the liver tissue (10 mg) was homogenized in 100 μL of water on ice, and the homogenates were boiled for 5 min and centrifuged at 13,000× *g* for 5 min. Glycogen content was measured using a quantification kit (Sigma-Aldrich, St. Louis, MO, USA). In addition, to measure the TG contents, the liver tissue (100 mg) was homogenized by adding 1 mL of 5% Nonidet-P40 substitute. The homogenate was heated in a water bath (80–100 °C) for 5 min and then cooled at room temperature, and the heating procedure was repeated once more to solubilize all TG. The homogenate was centrifuged at 2800× *g* for 2 min to remove insoluble materials, and the supernatant was diluted 10 times before analysis. The TG content was measured using a quantification kit (Sigma-Aldrich, St. Louis, MO, USA). The glycogen and TG levels were quantified by the protein concentration of the lipid extract as measured using the Bradford analysis.

### 2.14. Western Blot Analysis

The expression levels of insulin-signaling proteins in liver tissue were determined by Western blot analysis. Briefly, tissues were lysed with RIPA buffer containing a protease and phosphatase inhibitor cocktail. The homogenous solution was centrifuged at 13,000 rpm for 20 min at 4 °C and obtained from the supernatant. Protein levels in lysates were quantified using the Bradford protein assay (Bio-Rad, Hercules, CA, USA). The protein (20 μg) was mixed with loading buffer, electrophoresed on a 10% sodium dodecyl sulfate-polyacrylamide gel, and transferred to a polyvinylidene difluoride membrane. Proteins were blocked with 5% skim milk for 60 min, and membranes were incubated overnight with primary antibodies against p-IRS-1, IRS-1, p-PI3K, PI3K, p-Akt, and Akt (1: 1000) or GAPDH (1: 2000) in 5% skim milk at 4 °C. Membranes were washed three times with tris buffered saline containing Tween-20 (TBST), further reacted with goat anti-rabbit IgG-HRP conjugated secondary antibody (1:2000) at room temperature for 90 min, and washed three times with TBST buffer. Proteins were visualized using the ECL detection kit (Amersham Pharmacia, Piscataway, NJ, USA) and quantified using the Image J program 1.3 (National Institute of Health, Bethesda, MD, USA).

### 2.15. Statistical Analysis

All data are expressed as mean ± standard deviation (SD) of the mean of triplicate determinants. All statistical analyses were performed using Prism software (GraphPad Software, San Diego, CA, USA). One-way analysis of variance (ANOVA) was used for comparisons among groups. Significant differences between the mean values were assessed using Tukey’s multiple comparison test. *P*-values < 0.05 were considered to indicate statistical significance.

## 3. Results

### 3.1. Glucose Uptake and Glycogen Content on the PA-Treated HepG2 Cells

**Figure 1:**
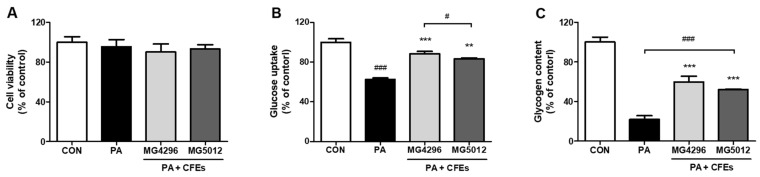
Effect of MG4296 and MG5012 on (**A**) cell viability, (**B)** glucose uptake, and (**C**) glycogen contents in insulin resistant-induced HepG2 cells by palmitic acid. Data are presented as mean ± SD (*n* = 3). ** *p <* 0.01, *** *p <* 0.001 vs. CON cells, and # *p <* 0.05, ### *p <* 0.001 vs. PA-treated cells.

The cytotoxicity of the CFEs of both strains on HepG2 cells was evaluated using the MTT assay. CFEs treatment of both strains at 10^9^ CFU/mL in PA-treated HepG2 cells did not affect the cell viability (Figure 1A). In the present study, IR in HepG2 cells was induced by PA treatment.

In addition, glucose uptake and glycogen content were measured to determine whether MG4296 and MG5012 could improve IR in PA-treated hepatocytes. In Figure 1B, IR-induced cells showed significantly decreased glucose uptake compared to control cells, while MG4296 and MG5012 CFEs-treated cells showed 25.9% and 20.8% higher glucose uptake than IR-induced cells. PA treatment also significantly reduced the glycogen content in HepG2 cells (Figure 1C). In contrast, the glycogen content of MG4296- and MG5012-treated cells was 37.9% and 30.1% higher than that of IR-induced cells (*p <* 0.001), respectively.

### 3.2. Changes of Body Weight, Liver, and Adipose Tissues Weights

**Figure 2:**
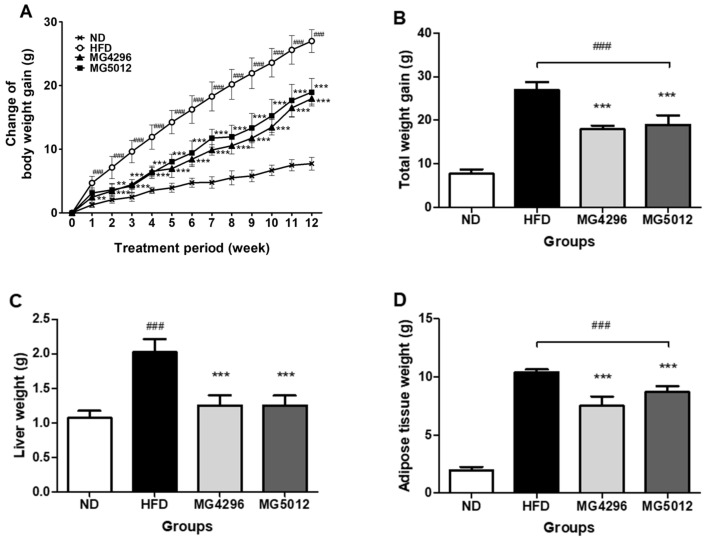
Effect of MG4296 and MG5012 on (**A**) body weight gain, (**B**) total weight gain, (**C**) liver weight, and (**D**) adipose tissue weight in HFD-induced T2DM mice. Mice were fed with ND or HFD for 12 weeks. HFD-fed mice were treated with an experimental vehicle or both strains. Body weight was measured weekly. Data are presented as mean ± SD (*n* = 6). ### *p <* 0.001 vs. ND group. ** *p <* 0.01, *** *p <* 0.001 vs. HFD group. ND; normal diet, HFD; high-fat diet.

Body weight was observed to assess weight change in HFD-fed mice (Figure 2A,B). After 12 weeks of the HFD-sugar water supply, the weight gain of the HFD group (27.0 ± 1.8 g) was significantly higher than that of the ND group (7.7 ± 1.0 g) (*p <* 0.001). While MG4296 (17.95 ± 0.84 g) and MG5012 (18.97 ± 2.17 g) groups showed significantly reduced body weight gain by 33.5% and 29.7%, respectively, compared to the HFD group (Figure 2B).

In addition, the differences in the weights of liver and AT in mice were examined. The liver weight of the ND group was approximately 1.08 ± 0.10 g, and that of the HFD group increased 2-fold to 2.02 ± 0.19 g (Figure 2C). The liver weights of MG4296 (1.25 ± 0.15 g) and MG5012 (1.25 ± 0.14 g) were significantly lower than that of the HFD group. The adipose tissue weight of mice in the HFD group was significantly higher than that of the ND group (*p* < 0.001). The AT weight of the strain administration groups was significantly reduced by 27.7% and 16.4%, respectively, compared to the HFD group (Figure 2D).

### 3.3. Effect of MG4296 and MG5012 on the IR

**Figure 3:**
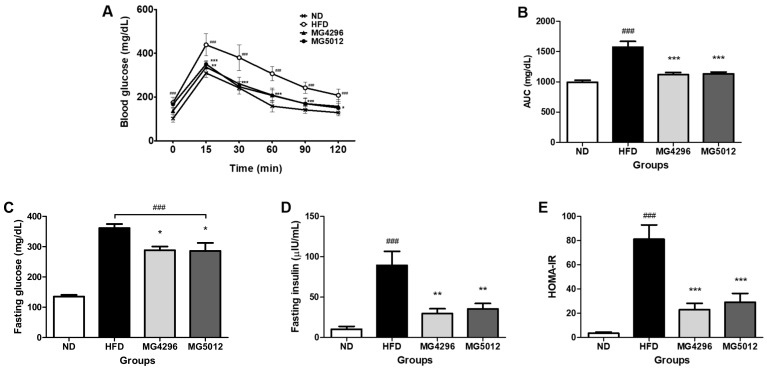
Effect of MG4296 and MG5012 on (**A**) oral glucose tolerance test (OGTT), (**B**) area under the curve (AUC), (**C**) fasting glucose, (**D**) fasting insulin, and (**E**) homeostatic model assessment of insulin resistance (HOMA-IR) in HFD-induced T2DM mice. Data are presented as mean ± SD (*n* = 6). ### *p <* 0.001 vs. ND group. * *p <* 0.05, ** *p <* 0.01, *** *p <* 0.001 vs. HFD group. HFD group. ND; normal diet, HFD; high-fat diet.

After 12 weeks of treatment, OGTT was used to estimate the relative role of insulin secretion, and the results are shown in Figure 3A. Blood glucose levels increased significantly at 15 min after glucose administration. However, the MG4296 and MG5012 administered groups had significantly lower blood glucose levels than the HFD group at 30, 60, and 90 min. The glucose levels in all groups returned to near-fasting levels after 120 min. The AUC value for the OGTT result was also significantly higher in the HFD group than in the ND group and significantly reduced by both strains treated groups (Figure 3B).

In addition, fasting glucose and insulin levels were measured to evaluate the HOMA-IR of both strains (Figure 3C,D). Fasting glucose and insulin were significantly increased in both the HFD group; however, these increased levels were remarkably reduced in both strains administrated groups. The HOMA-IR levels were significantly elevated in response to the HFD group compared to the ND group (*p <* 0.001). In contrast, MG4296 and MG5012 administration reduced the increased HOMA-IR index by more than 64% compared to the HFD group (Figure 3E).

### 3.4. Effect of MG4296 and MG5012 on Serum Profiles

**Figure 4:**
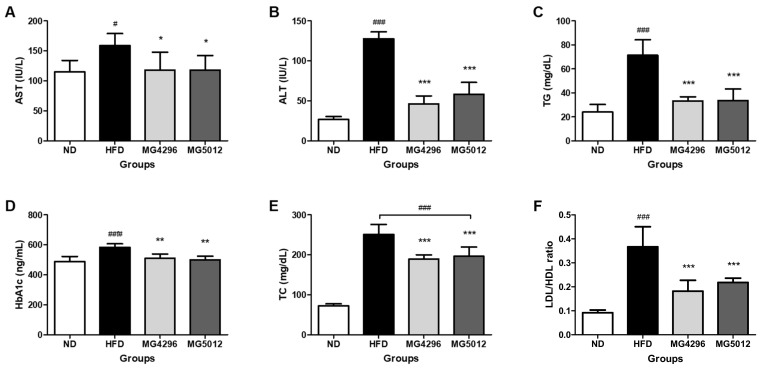
Biochemical profile of serum in HFD-induced T2DM mice. (**A**) AST, (**B**) ALT, (**C**) TG, (**D**) HbA1c, (**E**) TC, and (**F**) LDL/HDL ratio. Data are presented as mean ± SD (*n* = 6). # *p <* 0.05, ### *p <* 0.001 vs. ND group. * *p* < 0.05, ** *p <* 0.01, *** *p <* 0.001 vs. HFD group. ND; normal diet, HFD; high-fat diet.

After 12 weeks of treatment, changes in serum biochemical parameters in mice were measured. In Figure 4, the HFD-fed group showed significantly increased levels of AST, ALT, HbA1c, and lipid-related biomarkers (TG, TG, and LDL/HDL ratio) compared to the ND group. On the other hand, the MG4296 and MG5012 administration significantly alleviated the serum profiles increased by the HFD supplement. The AST levels of both strains administered groups were similar to that of ND group. ALT levels were decreased by 63.8% and 54.5% compared to the HFD group (*p* < 0.001) (Figure 4A,B).

In addition, the TG levels of the HFD group (71 ± 12 mg/dL) were significantly higher than those of the ND group (24 ± 6 mg/dL) and decreased by over 50% in the MG4296 and MG5012 groups (Figure 4C). HbA1c reflects the average plasma glucose concentration over a period [30]. HbA1c levels increased significantly in the HFD group compared to the ND group (*p* < 0.05), and those of the MG4296 and MG5012 groups decreased significantly compared to the HFD group (*p* < 0.01) (Figure 4D). Moreover, the TC and LDL/HDL ratios were also significantly lower in the LAB-administered groups than in the HFD group (*p* < 0.001) (Figure 4E,F).

### 3.5. Effect of MG4296 and MG5012 on Steatosis, Glycogen, and TG Content in Liver Tissues

**Figure 5:**
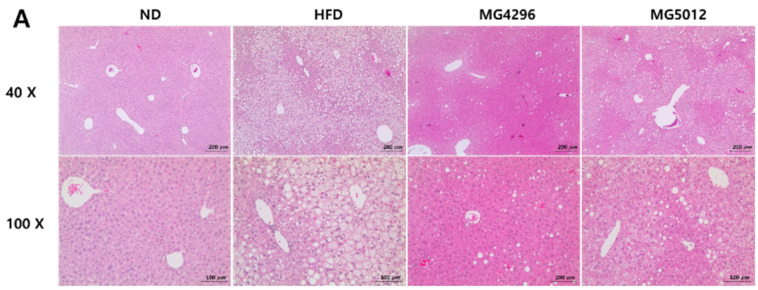

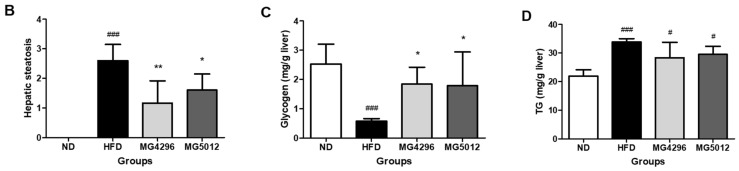
Histological changes in liver tissue of HFD-induced T2DM mice. (**A**) Representative photographs of hematoxylin and eosin (H&E)-stained sections of liver tissues (40× and 100× magnification), (**B**) Hepatic steatosis score, (**C**) Glycogen content, (**D**) Triglyceride (TG) content. Results are presented as mean ± SD (*n* = 6). # *p <* 0.05, ### *p <* 0.001 vs. ND group. * *p <* 0.05, ** *p <* 0.01 vs. HFD group. ND; normal diet, HFD; high-fat diet.

After 12 weeks of treatment, the effect of both strains on hepatic steatosis was evaluated by analyzing histopathological changes in the liver tissue stained with H&E. As shown in Figure 5A, fat accumulation in the cytoplasm of hepatocytes was observed as a vacuole with a clear boundary under a microscope. However, in the present model, no noticeable inflammatory lesions were found in any of the groups. Hepatic steatosis remarkably increased in the HFD group (2.60 ± 0.55) compared to the ND group. On the other hand, it was significantly decreased in the MG4296 (1.17 ± 0.75) (*p <* 0.001) and MG5012 (1.60 ± 0.55) (*p <* 0.01) group compared to the HFD group. Liver weights were similar in both groups; however, fat accumulation was lower in the MG4296 group than in the MG5012 group (Figure 5B).

The glycogen level in liver tissues was significantly reduced in the HFD group compared to the ND group (*p <* 0.001); however, the glycogen levels in the MG4296 and MG5012 groups were significantly higher than those in the HFD group (*p <* 0.05) (Figure 5C). On the other hand, increased TG levels due to HFD supply were also significantly decreased by MG4296 and MG5012 administration (Figure 5D).

### 3.6. Effect of MG4296 and MG5012 on Ameliorating IR through the PI3K/Akt Pathway in Liver Tissues

**Figure 6:**
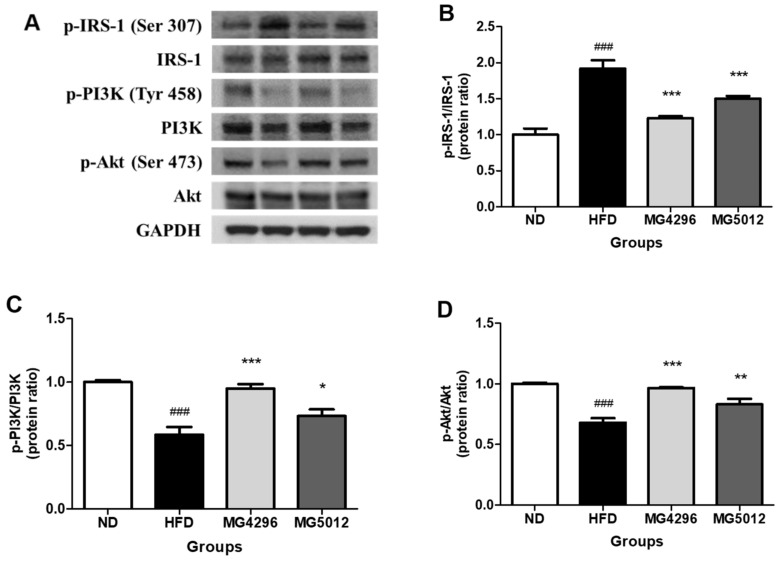
Effects of MG4296 and MG5012 on the IRS-1/PI3K/Akt protein expression in liver tissue of HFD-induced T2DM mice. (**A**) Representative western blot, (**B**) p-IRS-1/IRS-1, (**C**) p-PI3K/PI3K, and (**D**) p-Akt/Akt. Data are presented as mean ± SD (*n* = 3). ### *p <* 0.001 vs. ND group. * *p <* 0.05, ** *p <* 0.01, *** *p <* 0.001 vs. HFD group. ND; normal diet, HFD; high-fat diet.

To confirm the change in IR by administration of MG496 and MG5012 in liver tissues of HFD-induced T2DM mice, we measured the expression levels of IRS-1, PI3K, and Akt, involved in the insulin signaling pathway. The expression patterns of these genes are presented in Figure 6A. The HFD group showed significantly upregulated phosphorylation of IRS-1 (p-IRS-1/IRS-1 ratio) compared to the ND group. However, the phosphorylation of IRS-1 in the MG4296 and MG5012 groups was 35.9% and 21.7% lower than that in the HFD group, respectively (*p <* 0.001) (Figure 6B). On the other hand, phosphorylation of PI3K (p-PI3K/PI3K ratio) and Akt (p-Akt/Akt ratios) was lower in the HFD group than in the ND group. However, the expression levels of p-PI3K and p-Akt were higher in the MG4296 and MG5012 groups than in the HFD group (Figure 6C,D).

## 4. Discussion

T2DM is a complex disease characterized by excessive glucose production in the liver, IR, and decreased glucose processing capacity in muscles and adipocytes [30]. T2DM and metabolic syndrome are closely correlated with an increase in the obese population. IR causes a decrease in glucose influx in muscle cells, a decrease in glucose synthesis inhibition in hepatocytes, and an excessive increase in glucose, resulting in an increase in blood sugar and an excessive influx of free fatty acids into the blood [31]. Free fatty acids decomposed from adipocytes damage the insulin signaling pathway, thereby lowering the sensitivity of the insulin receptors that bind to insulin even if insulin is secreted from the pancreas [32].

Recently, probiotics have gained attention as an important source for treating and preventing metabolic syndrome [33,34]. In particular, several studies have suggested that probiotics can alleviate the symptoms of diabetes and prevent complications caused by diabetes. When *L. rhamnosus* GG was administered to KK-Ay mice, a T2DM mouse model, fasting blood glucose levels were significantly reduced [35]. *L. plantarum* No. 14 prevented the development of IR by lowering serum leptin and insulin levels and reducing the accumulation of white adipose tissue [36]. *L. paracasei* TD062 attenuates T2DM by regulating the insulin signaling pathway and glucose metabolism in diabetic mice [37].

In a previous study, we identified two strains (MG4296 and MG5012) as probiotics candidates with antidiabetic effects via α-glucosidase inhibition and antioxidative effects in vitro. Therefore, in the present study, we confirmed the improvement effect and mode of action of both strains on T2DM using in vitro and in vivo IR-induced models.

IR in hepatocytes primarily causes impaired glycogen synthesis and fails to inhibit glucose production, a major cause of hyperglycemia [38]. HepG2 cells are used extensively to study the regulation of hepatic glucose production and insulin pathways as a cell culture model of human hepatocytes [39,40]. In this study, we evaluated the potential protective effects of MG4296 and MG5012 against PA stimulus-induced insulin signaling in HepG2 cells. Glucose consumption and glycogen production in IR-induced HepG2 cells were significantly improved by MG4296 and MG5012 administration. In a previous study, *L. paracasei* 1F-20 in IR-induced HepG2 cells increased glucose uptake [41]. *L. plantarum* Q180 alleviated hypertriglyceridemia by inhibiting lipid synthesis in PA-induced HepG2 cells [42]. These results suggest that MG4296 and MG5012 could improve IR in HepG2 cells by increasing glucose uptake and glycogen content.

IR accompanying obesity is considered to be the most important antecedent of T2DM and a key factor in the occurrence of metabolic syndrome [43]. Thus, long-term intake of HFD leads to increased fat cells and IR. T2DM occurs when obese-induced mice have moderate glucose intolerance and increased IR [44]. Previous studies have also shown that an increase in visceral fat over subcutaneous fat increases hyperinsulinemia, IR, and the incidence of metabolic complications, such as hypertension and dyslipidemia [45]. When IR is triggered, the liver produces and secretes excess glucose and improperly produces lipids due to hyperinsulinemia [46]. Thus, excessive lipid accumulation in the liver and adipose tissue is indicated by increased tissue weight. In this study, the antidiabetic efficacy of MG4296 and MG5012 was evaluated in a T2DM C57BL/6 mouse model prepared by feeding HFD and sugar water for 12 weeks. We observed that MG4296 and MG5012 administration significantly reduced the body weight gain and liver and AT weights increased by HFD supplementation in T2DM mice. Similar to our results, Balakumar et al. [16] reported that *L. plantarum* MTCC5689 significantly reduced body weight gain in T2DM mice. Hsieh et al. [6] reported that *L. reuteri* GMNL-263 in T2DM rats reduced liver tissue weight. These results demonstrated that the obesity mice model in this study was effectively induced by high fat and sugar supplement and that the administration of MG4296 and MG5012 had the effect of significantly reducing the weight gain in an obese mouse by inhibiting the production of fat in the liver and adipose tissues.

IR is an important indicator of T2DM because it promotes hyperglycemia and increases the risk of metabolic diseases [47]. Clinically, elevated blood insulin levels during fasting are used as an indicator of IR. HOMA-IR is a method of evaluating insulin receptor homeostasis by measuring fasting blood glucose and insulin levels and is currently widely used as an estimate of insulin sensitivity [48]. In this study, we evaluated the effects of both strains on the IR of TD2M-induced mice. The OGTT, AUC, fasting glucose, fasting insulin, and HOMA-IR levels were significantly elevated by high-fat and sugar-water diets, while they were significantly reduced by the administration of the two probiotic strains. In a previous study, *L. plantarum* CCFM0236 showed improved glucose tolerance by reducing HOMR-IR (59%) in T2DM mice [49]. In addition, some strains of *L. paracasei* have been reported to reduce IR in diabetic mice [50]. Therefore, it was suggested that both MG4296 and MG5012 could improve IR by significantly reducing glucose tolerance and HOMA-IR.

T2DM patients have an abnormal blood lipid profile. Increased serum AST and ALT levels are associated with IR [51,52]. In addition, plasma levels of TG, TC, and LDL were significantly higher than those of normal subjects [53]. In this study, blood glucose and lipid profiles were effectively improved in T2DM-induced mice by MG4296 and MG5012 administration. Therefore, these results demonstrate the important characteristics of these potential probiotics in improving relevant serum markers in diabetes management.

The liver plays an important role in glucose–lipid metabolism and energy homeostasis, which controls blood sugar levels. Diabetes is accompanied by various complications and is considered a major risk factor for hepatic dysfunction [54]. Hepatic dysfunction includes IR, impaired glucose tolerance, and transient or chronic glucose metabolism disorders. In particular, glycogen and TG levels in liver tissue strongly correlate with IR [55]. Gastaldelli et al. [56] reported that hepatic TG levels in T2DM patients are directly related to the severity of IR in the liver. When IR reduces insulin stimulation, glycogen synthesis and glucose transport activity are also reduced. DM and NAFLD intersect in many areas. The prevalence of NAFLD in DM patients is about 50%-70%, which is approximately twice as common in nondiabetic groups. The underlying causes of fatty liver are obesity and IR. The leading cause of NAFLD is increased fatty acid outflow into the liver, facilitated by the high solubility of plasma-free fatty acids (FFA), which ultimately leads to the accumulation of TG in the liver tissue. In this study, histological changes in liver tissue were confirmed in T2DM-induced mice. Steatosis and TG accumulation in liver tissue were reduced, and glycogen content in liver tissue was increased by administering MG4296 and MG5012. In a previous study, *L. plantarum* FZU3013 improved NAFLD and hyperlipidemia in mice fed an HFD [57]. *L.* GG improved hepatic steatosis in HFD-induced mice [58]. In accordance with previous studies, the present study confirmed that the administration of both strains improved IR by increasing glycogen synthesis and inhibiting lipid accumulation in terms of histopathology. These results indicated that MG4296 and MG5012 administration could protect fat accumulation in the liver in the early stages of NAFLD induced by a high fat and sugar diet.

The direct cause of IR by obesity is the accumulation of fat in the cells of muscle and liver tissues, which are important for glucose metabolism. The PI3K/Akt signaling pathway is a classic route of IR [59]. Glucose transport in the liver is initiated by the phosphorylation of the insulin-dependent pathway, insulin receptor substrate-1 (IRS-1), and binds PI3K to be active in the sub-protein Akt. The latter, activated by PI3K, inhibits glucose degradation and gluconeogenesis, thereby inhibiting glucose production. IRS-1 serine phosphorylation suppresses insulin signaling by reducing tyrosine phosphorylation, thereby suppressing downstream effectors in insulin signaling, such as PI3K and Akt [60]. In the present study, the administration of MG4296 and MG5012 resulted in the downregulation of p-IRS-1 and upregulation of p-PI3k and p-Akt. Similarly, the intake of *L. casei* CCFM419 in T2DM mice improved IR through PI3K/Akt regulation [48]. These results suggest that MG4296 and MG5012 exhibit antidiabetic effects by restoring the insulin signaling pathway.

## 5. Conclusions

The present study confirmed the improvement of the IR and antidiabetic effects *of L. plantarum* MG4296 and *L. paracasei* MG5012 in insulin-resistant HepG2 cells in vitro and in an HFD-induced T2DM mouse model in vivo. Both strains increased glucose uptake and glycogen content in IR HepG2 cells. They also significantly reduced weight gain, hepatic steatosis and significantly improved serum profiles, including ALT, HBA1c, TG, and LDL/HDL levels. In addition, these strains improved IR by increasing glucose tolerance and reducing HOMA-IR levels. Both strains have been identified to enhance IR through the PI3K/Akt signaling pathway. Therefore, we suggest that *L. plantarum* MG4296 and *L. paracasei* MG5012 could be used as potential probiotics to ameliorates T2DM.

## Data Availability

The data presented in this study are available on request from the corresponding authors.
